# A checklist of the marine Anthuroidea (Crustacea: Isopoda: Cymothoida) from the reefs of Peninsular Malaysia, with some new distributional data

**DOI:** 10.3897/BDJ.8.e54748

**Published:** 2020-06-26

**Authors:** Melvin Chew, Azman Abdul Rahim

**Affiliations:** 1 Department of Earth Sciences and Environment, Faculty of Science and Technology, Universiti Kebangsaan Malaysia, 43600 UKM, Bangi, Selangor, Malaysia Department of Earth Sciences and Environment, Faculty of Science and Technology, Universiti Kebangsaan Malaysia, 43600 UKM, Bangi Selangor Malaysia; 2 Marine Ecosystem Research Centre (EKOMAR), Faculty of Science & Technology, Universiti Kebangsaan Malaysia, 43600 UKM, Bangi, Selangor, Malaysia Marine Ecosystem Research Centre (EKOMAR), Faculty of Science & Technology, Universiti Kebangsaan Malaysia, 43600 UKM Bangi, Selangor Malaysia

## Abstract

**Background:**

An up-to-date checklist of the Peninsular Malaysian marine Anthuroidea is presented, based on previous taxonomic or ecological literature and recent collections. The present study, a part of the subproject on the Biodiversity of Sultan Iskandar Marine Park, recognised 24 species in 12 genera and 5 families from Peninsular Malaysia. An extensive list of bibliographical references, detailed information on habitat and distributional records, museum locations of type material are provided for each species. Amongst the listed species, 11 are recently discovered Malaysian species belonging to the genera *Amakusanthura* Nunomura, 1977, *Apanthura* Stebbing, 1900, *Expanathura* Wägele, 1981, *Leptanthura* G. O. Sars, 1897, *Kupellonura* Barnard, 1925, *Pendanthura* Menzies & Glynn, 1968 and *Tinggianthura* Chew, Rahim & bin Haji Ross, 2014. Our records were limited to shallow subtidal reefs of peninsular Malaysian coast, suggesting that the number of species in the list may rise with an extensive survey.

**New information:**

The up-to-date checklist of marine Anthuroidea of the Peninsular Malaysia comprises 24 species in 12 genera and 5 families, including some new distributional data.

## Introduction

Tropical reefs are known to support enormous biodiversity and portray a high priority for conservation action amongst marine ecosystems (Roberts 2002). A faunal inventory of our planet remains an urgent task. A basic taxonomic knowledge is necessary for bio-evolutionary studies, as well as for understanding biogeography. The Isopoda is one of the most successful and rapidly evolving malacostracan orders, displaying a tremendous diversity in marine, terrestrial and continental waters. Although isopods have colonised almost every niche and are the most important group in terms of specific diversity, they have been largely ignored in studies dealing with conservation strategies. According to Wilson (2007), the isopods contain over 10,300 species that are primarily marine, but they can also be found in brackish water and freshwater environments. In addition, more than 3,600 terrestrial species of isopods are known (Schmalfuss 2003). Isopods play an important role in marine food chains due to their scavenging nature in benthic communities Keable (2006). Placed strategically within the Sundaland region of biodiversity hotspot and the coral Indo-Malaysian triangle (Hoeksema 2007, Myers et al. 2000), Malaysia is expected to hold a high diversity of isopods, particularly the anthuroids. The aim of this work is to update the knowledge the anthuroids inhabiting the shallow water reef of the Malaysian Peninsular (MP).

## Materials and methods

**Figure 1.**
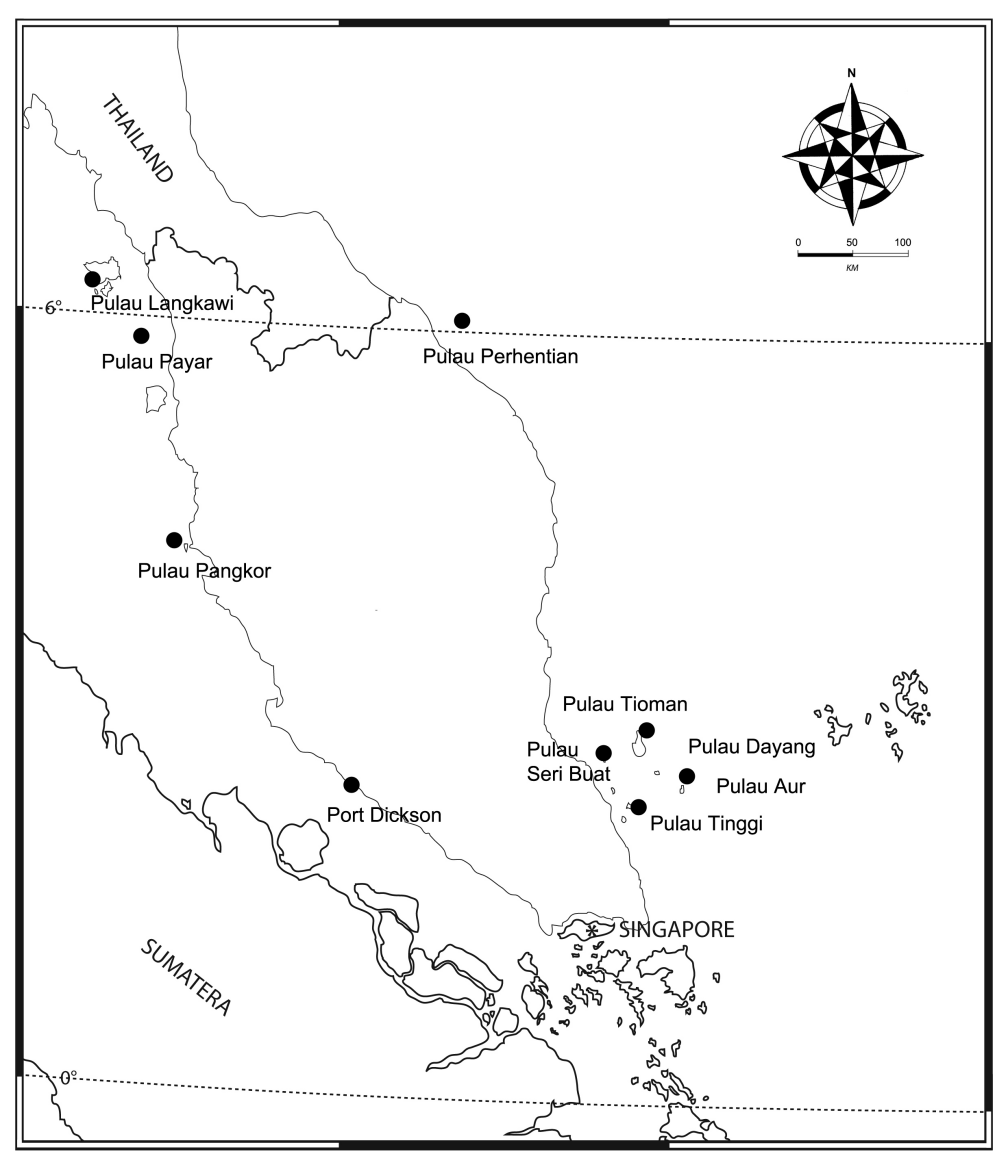
Map of collection localities in the Malaysian Peninsular.

The material collected was obtained from both west and east coasts of the Peninsular Malaysia. The following collection localities were from the west coast of MP: Pulau Langkawi (6°21'56.05"N, 99°40'31.13"E), Pulau Payar (6°03'23.0"N, 100°01'55.0"E), Pulau Pangkor (4°11'22.32"N, 100°32'50.22"E) and Port Dickson (2°24'57.0"N, 101°51'10.3"E). The following collection localities were from the east coast of MP: Pulau Perhentian (5°52'59.48"N, 102°44'31.66"E), Pulau Tioman (2°54'15.44''N, 104°6'1.08''E), Pulau Seri Buat (2°41'13.59"N, 103°55'25.99"E), Pulau Aur (2°28'17.24"N, 104°30'53.14"E), Pulau Dayang (2°28'40.90"N, 104°30'19.12"E) and Pulau Tinggi (2°17'37.96"N, 104°6'1.97"E) (Fig. 1). See Chew et al. (2016) for an explanation of the biological material collection and Chew et al. (2018) for the identification and morphological study procedures. The identification was done using the specialised literature and identification keys from Kensley (1980),Kensley and Poore (1982),Negoescu 1997,Kensley (2001) and Poore and Lew Ton (2002). Only primary synonyms are included for each species under the "Nomenclature" field. The checklist of the marine anthuroids from MP included an updated list of materials, habitat and distribution records. Materials are located in the following museums: Muzium Zoologi, Universiti Kebangsaan Malaysia (UKMMZ); Lee Kong Chian Natural History Museum, National University of Singapore (ZRC); Museum d’Histoire Naturelle, Genève (MHNG); Zoologisches Museum Berlin (ZMB); and Muséum National d'Histoire Naturelle, Paris (MNHN).

## Data resources

All Anthuroidea records were cross-checked for their taxonomy in the World Register of Marine Species (WoRMS Editorial Board 2020).

## Checklists

### Checklist of marine Anthuroidea known to occur in Peninsular Malaysia

#### Amakusanthura koonyumae

Bamber, 1997

7AEC1724-0A86-581F-8779-20323CF5582E

http://www.marinespecies.org/aphia.php?p=taxdetails&id=255360

Amakusanthura koonyumae : 81–85, figs. 3-5; Bamber 2008: 860–861.

##### Materials

- **Type status:** Other material. **Location:** island: Labas, Pulau Tioman; country: Malaysia; stateProvince: Pahang; verbatimCoordinates: N2°53'13.71", E104°3'54.65"; **Event:** eventDate: 18 April 2014; habitat: In fine sand to medium sand from around ‘*Cerianthus*’ at 2 to 3 m depth (Bamber, 1997); amongst coral rubble. Littoral 0.5 to 15 m depth (present study); eventRemarks: C. Melvin; **Record Level:** collectionCode: (1 female) UKMMZ-1571

##### Distribution

HONG KONG―Tolo Channel (type locality), Conic Island Cave. MALAYSIA―Pahang State: Pulau Tioman (new record).

##### Notes

Fig. 2a

#### Apanthura bruscai

Müller, 1992

90B7893E-BE01-5EAE-A2FF-59F056823BC0

http://www.marinespecies.org/aphia.php?p=taxdetails&id=255383

Apanthura bruscai Müller 1992b: 156–161, figs. 1–19.

##### Materials

- **Type status:** Holotype. **Location:** island: Pulau Babi Besar; country: Malaysia; stateProvince: Johor; verbatimCoordinates: N2°50.483', E104°9.566'; **Event:** habitat: Fringing-reef and reef-flat under coral rocks mainly covered with coralline algae, intertidal (Müller 1992a); **Record Level:** modified: male, 3.4 mm; datasetID: ZMB 26953

##### Distribution

Only known from type locality (Pulau Babi Besar, east coast Peninsular Malaysia).

##### Notes

This species record is based on literature only. No specimen was collected during the present study.

#### Apanthura pariensis

Negoescu, 1997

1717BC2B-3042-50A1-850A-4EC97FD0B98C

http://www.marinespecies.org/aphia.php?p=taxdetails&id=255402

MF680510

Apanthura pariensis Negoescu 1997: 186–194, figs. 6–10; Chew et al. 2018: 74–75, fig. 1.

##### Materials

- **Type status:** Other material. **Location:** island: Pulau Pangkor; country: Malaysia; stateProvince: Perak; verbatimCoordinates: N4°11'22.32", E100°32'50.22"; **Event:** eventDate: 15 Apr 2014; habitat: Littoral 0.5 to 3 m depth, amongst coral rubble (Chew et al. 2018); **Record Level:** collectionCode: (1 female) UKMMZ-1583; (1 male) UKMMZ-1584; (43 females, 2 males) UKMMZ-1585
- **Type status:** Other material. **Location:** island: Pulau Langkawi; country: Malaysia; stateProvince: Kedah; verbatimCoordinates: N6°21'56.05" E99°40'31.13"; **Event:** eventDate: 4 Nov 2013; habitat: Intertidal, amongst coral rubble; **Record Level:** collectionCode: (9 females) UKMMZ-1586
- **Type status:** Other material. **Location:** island: Pulau Langkawi; country: Malaysia; stateProvince: Kedah; verbatimCoordinates: N6°21'56.05" E99°40'31.13"; **Event:** eventDate: 8 Mac 2015; habitat: Intertidal, amongst coral rubble; **Record Level:** collectionCode: (8 females, 2 males) UKMMZ-1587

##### Distribution

INDONESIA―Pari Island, Java Sea (type locality). MALAYSIA―Perak State: Pulau Pangkor (new record).―Kedah State: Pulau Langkawi (new record).

##### Notes

Fig. 2b

#### Apanthura stocki

(Müller, 1991)

6D116825-BF79-58DD-A5B2-6C9FFB7E06AF

http://www.marinespecies.org/aphia.php?p=taxdetails&id=255410

MF680509

Amakusanthura stocki Müller 1991: 595–600, figs. 30–56.
Apanthura stocki Müller 1992b: 166; Chew et al. 2018: 75-76, fig. 2.

##### Materials

- **Type status:** Other material. **Occurrence:** recordedBy: C. Melvin; **Location:** island: Mentinggi, Pulau Tinggi; country: Malaysia; stateProvince: Johor; verbatimCoordinates: N2°16'21.67", E104°7'18.61"; **Event:** eventDate: 19 April 2013; habitat: Littoral 0.5 to 3 m depth, amongst coral rubble (Chew et al. 2018); **Record Level:** collectionCode: (1 female, 1 male) UKMMZ-1576
- **Type status:** Other material. **Occurrence:** recordedBy: C. Melvin; **Location:** island: Kg Pasir Panjang, Pulau Tinggi; country: Malaysia; stateProvince: Johor; verbatimCoordinates: N2°17'37.96", E104°6'1.97"; **Event:** eventDate: 18 Dec 2012; habitat: Littoral 0.5 to 3 m depth, amongst coral rubble (Chew et al. 2018); **Record Level:** collectionCode: (3 females, 1 male) UKMMZ-1577
- **Type status:** Other material. **Occurrence:** recordedBy: C. Melvin; **Location:** island: Sebirah Kechil, Pulau Tinggi; country: Malaysia; stateProvince: Johor; verbatimCoordinates: N2°18.622', E104°05.616'; **Event:** eventDate: 18 Apr 2013; habitat: Littoral 0.5 to 3 m depth, amongst coral rubble (Chew et al. 2018); **Record Level:** collectionCode: (14 females) UKMMZ-1578
- **Type status:** Other material. **Occurrence:** recordedBy: C. Melvin; **Location:** island: Pulau Seri Buat; country: Malaysia; stateProvince: Pahang; verbatimCoordinates: N2°41'13.59", E103°55'25.99"; **Event:** eventDate: 19 Apr 2014; habitat: Littoral 0.5 to 7 m depth, amongst coral rubble (Chew et al. 2018); **Record Level:** collectionCode: (1 male) UKMMZ-1579
- **Type status:** Other material. **Occurrence:** recordedBy: C. Melvin; **Location:** island: Kg Pasir Panjang, Pulau Tinggi; country: Malaysia; stateProvince: Johor; verbatimCoordinates: N2°17'37.96", E104°6'1.97"; **Event:** eventDate: 15 Jun 2015; habitat: Littoral 0.5 to 3 m depth, amongst coral rubble (Chew et al. 2018); **Record Level:** collectionCode: (4 females) UKMMZ-1580
- **Type status:** Other material. **Occurrence:** recordedBy: C. Melvin; **Location:** island: Pulau Dayang; country: Malaysia; stateProvince: Johor; verbatimCoordinates: N2°28'40.90", E104°30'19.12"; **Event:** eventDate: 26 Jul 2016; habitat: Littoral 0.5 to 3 m depth, amongst coral rubble (Chew et al. 2018); **Record Level:** collectionCode: (27 females) UKMMZ-1581
- **Type status:** Other material. **Occurrence:** recordedBy: C. Melvin; **Location:** island: Pulau Aur; country: Malaysia; stateProvince: Johor; verbatimCoordinates: N2°28'17.24", E104°30'53.14"; **Event:** eventDate: 26 Jul 2016; habitat: Littoral 0.5 to 3 m depth, amongst coral rubble (Chew et al. 2018); **Record Level:** collectionCode: (6 females) UKMMZ-1582

##### Distribution

SRI LANKA―Indian Ocean (type locality); MALAYSIA―Johor State: Pulau Besar; Pulau Tinggi; Pulau Seri Buat (new record).

##### Notes

Fig. 2c

#### Apanthura tiomanae

Müller, 1992

48B4900A-914E-5D55-8559-2B11DD147ABF

http://www.marinespecies.org/aphia.php?p=taxdetails&id=255413

Apanthura tiomanae Müller 1992b: 161–166, figs 20–39.

##### Materials

- **Type status:** Other material. **Occurrence:** recordedBy: C. Melvin; **Location:** island: Pulau Tioman; country: Malaysia; stateProvince: Pahang; verbatimCoordinates: N2°54'15.44", E104° 6'1.08"; **Event:** eventDate: 18 April 2014; habitat: Amongst coral rubble, littoral 0.5 to 7 m depth; **Record Level:** collectionCode: (1 female) UKMMZ-1572

##### Distribution

MALAYSIA―Pahang State: Pulau Tioman (type locality).

##### Notes

Fig. 2d

#### Cyathura bentotae

Müller, 1991

A1DC35BE-90E0-5F31-897B-C41D039A2F67

http://www.marinespecies.org/aphia.php?p=taxdetails&id=255423

Cyathura bentotae Müller 1991: 603–607, figs. 57–80; Müller 1992b: 166.

##### Materials

- **Type status:** Holotype. **Event:** habitat: Fringing-reef and sabellid reef; dead coral substratum, 1 to 3 m depth; **Record Level:** collectionCode: (male, 3.4 mm) ZMB 26953; 1 cr (ZMB 26956)

##### Distribution

SRI LANKA―Indian Ocean (type locality); MALAYSIA―Pahang State: Pulau Tioman.

##### Notes

This species record is based on literature only. No specimen was collected during the present study.

#### Mesanthura albolineata

Barnard, 1925

D8243EE3-31A8-5BF4-8F3E-CE91F61E8334

http://www.marinespecies.org/aphia.php?p=taxdetails&id=258320

Mesanthura albolineata Barnard 1925: 144, fig. 9c; Wägele 1984: 394–398, figs. 5–7; Negoescu and Wägele 1984: 125; Kensley 1987: 118; Müller 1993: 20.

##### Materials

- **Type status:** Holotype. **Location:** country: Singapore; **Event:** habitat: Fringing reef, outer reef-flat, reef-margin and upper coral-slope; dead coral substratum, *Acropora* sp. and *Pocillopora damicornis* (Linnaeus, 1758).; **Record Level:** collectionCode: (2 immature adults, 1 manca) ZMB 26938

##### Distribution

SINGAPORE―(type locality); MALAYSIA―Johor State: Pulau Babi Besar.

##### Notes

This species record is based on literature only. No specimen was collected during the present study.

#### Mesanthura asiatica

Müller, 1993

C91FB4BC-D900-5EF9-A294-F49DAD7B2F93

http://www.marinespecies.org/aphia.php?p=taxdetails&id=258321

Mesanthura asiatica : 20–25, figs. 1–25.

##### Materials

- **Type status:** Holotype. **Event:** habitat: Fringing reef, outer reef-flat, reef-margin and upper coral-slope; dead coral substratum, *Acropora* sp. and *Pocillopora damicornis* (Linnaeus, 1758); **Record Level:** collectionCode: (female, 4.0 mm) ZMB 26939a.
- **Type status:** Paratype. **Record Level:** collectionCode: (2 immature adults, 1 preparatory male; 1 manca) ZMB 26939b
- **Type status:** Other material. **Location:** island: Pulau Tioman; country: Malaysia; stateProvince: Pahang; verbatimCoordinates: N2°54'15.44", E104°6'1.08"; **Event:** eventDate: 18 April 2014; habitat: Amongst coral rubble, littoral 0.5 to 7 m depth; **Record Level:** collectionCode: (1 female) UKMMZ-1588; (1 male) UKMMZ-1589; (6 females, 1 male) UKMMZ-1590
- **Type status:** Other material. **Location:** island: Kg Pasir Panjang, Pulau Tinggi; country: Malaysia; stateProvince: Johor; verbatimCoordinates: N2°17'37.96", E104°6'1.97"; **Event:** eventDate: 16 Jun 2015; habitat: Amongst coral rubble, littoral 0.5 to 3 m depth; **Record Level:** collectionCode: (1 female) UKMMZ-1591

##### Distribution

MALAYSIA―Johor State: Pulau Babi Besar (type locality); Pulau Tinggi (new record);―Pahang State: Pulau Tioman (new record).

##### Notes

Fig. 2e

#### Mesanthura kiliani

Müller, 1993

62D3AD96-641F-5D52-A376-92B6FA074481

http://www.marinespecies.org/aphia.php?p=taxdetails&id=258340

Mesanthura kiliani Müller 1993: 25–29, figs. 26–42.

##### Materials

- **Type status:** Holotype. **Location:** island: Pulau Tioman; country: Malaysia; **Event:** habitat: Fringing reef, outer reef-flat, reef-margin and upper coral-slope; dead coral substratum, *Acropora* sp. and *Pocillopora damicornis*; **Record Level:** collectionCode: (female, 7.2 mm) ZMB 26940a
- **Type status:** Paratype. **Location:** island: Pulau Tioman; country: Malaysia; **Event:** habitat: Fringing reef, outer reef-flat, reef-margin and upper coral-slope; dead coral substratum, *Acropora* sp. and *Pocillopora damicornis*; **Record Level:** collectionCode: (2 postmancas, 2 mancas) ZMB 26940b.

##### Distribution

MALAYSIA―Pahang State: Pulau Tioman;―Johor State: Pulau Babi Besar.

##### Notes

This species record is based on literature only. No specimen was collected during the present study.

#### Mesanthura protei

Kensley, 1980

A525220C-59DC-51C9-9B6F-BB52C2E29BBC

http://www.marinespecies.org/aphia.php?p=taxdetails&id=211377

Mesanthura protei Kensley 1980: 30–32, figs. 22–23; Kensley and Poore 1982: 631–632, fig. 6; Wägele 1984: 748–752, figs. 8–10; Müller 1993: 33–39, figs. 61–99.

##### Materials

- **Type status:** Other material. **Location:** island: Batu Malang, Pulau Tioman; country: Malaysia; stateProvince: Pahang; verbatimCoordinates: N2°54'15.44", E104° 6'1.08"; **Event:** eventDate: 18 April 2014; habitat: Amongst coral rubble, littoral 0.5 to 7 m depth; **Record Level:** institutionCode: (1 female) UKMMZ-1592; (14 females) UKMMZ-1593

##### Distribution

MOZAMBIQUE―South of Inhambane (type locality); MADAGASCAR―Abrolhos Islands; THAILAND―Ko-Sichang; KENYA; MALAYSIA―Johor State: Pulau Babi Besar;―Pahang State: Pulau Tioman, (new record).

##### Notes

Fig. 2f

#### Mesanthura quadrata

Kensley & Schotte, 2000

68F3E9AF-BF62-5C43-A90F-7D82386902EB

http://www.marinespecies.org/aphia.php?p=taxdetails&id=211379

Mesanthura quadrata Kensley and Schotte 2000: 2080–2083, figs. 17–18.

##### Materials

- **Type status:** Other material. **Location:** island: Pulau Seri Buat; country: Malaysia; stateProvince: Pahang; verbatimCoordinates: N2°41'13.59", E103°55'25.99"; **Event:** eventDate: 19 April 2014; habitat: Amongst coral rubble, littoral 0.5 to 7 m depth; **Record Level:** collectionCode: (1 female) UKMMZ-1594, (3 immature females) UKMMZ-1595
- **Type status:** Other material. **Location:** island: Batu Malang, Pulau Tioman; country: Malaysia; stateProvince: Pahang; verbatimCoordinates: N2°54'15.44", E104°6'1.08"; **Event:** eventDate: 18 April 2014; habitat: Amongst coral rubble, littoral 0.5 to 7 m depth; **Record Level:** collectionCode: (1 immature female) UKMMZ-1596
- **Type status:** Other material. **Location:** island: Kg Pasir Panjang, Pulau Tinggi; country: Malaysia; stateProvince: Johor; verbatimCoordinates: N2°17'37.96", E104°6'1.97"; **Event:** eventDate: 15 June 2015; habitat: Amongst coral rubble, littoral 0.5 to 3 m depth; **Record Level:** collectionCode: (1 female) UKMMZ-1597
- **Type status:** Other material. **Location:** island: Sebirah Kechil, Pulau Tinggi; country: Malaysia; stateProvince: Johor; verbatimCoordinates: N2°18.622', E104°05.616'; **Event:** eventDate: 15 June 2015; habitat: Amongst coral rubble, littoral 0.5 to 3 m depth; **Record Level:** collectionCode: (1 female) UKMMZ-1598
- **Type status:** Other material. **Location:** island: Kg Pasir Panjang, Pulau Tinggi; country: Malaysia; stateProvince: Johor; verbatimCoordinates: N2°17'37.96", E104°6'1.97"; **Event:** eventDate: 13 Oct 2012; habitat: Assoc. artificial substrate unit at 3 m depth; **Record Level:** collectionCode: (29 juveniles) UKMMZ-1599
- **Type status:** Other material. **Location:** island: Sebirah Kechil, Pulau Tinggi; country: Malaysia; stateProvince: Johor; verbatimCoordinates: N2°18.622', E104°05.616'; **Event:** eventDate: 18 April 2013; habitat: Amongst coral rubble, littoral 0.5 to 3 m depth; **Record Level:** collectionCode: (1 female) UKMMZ-1560

##### Distribution

SEYCHELLES―Mahé beach, Mahé Island (type locality); MALAYSIA―Pahang State: Pulau Seri Buat, (new record); Pulau Tioman, (new record);―Johor State: Pulau Tinggi, (new record).

##### Notes

Fig. 2g

#### Pendanthura tinggiensis

Chew, bin Abdul Rahim & Mohd Yusof, 2016

08F7B939-508A-539F-9E42-66584690E57C

http://www.marinespecies.org/aphia.php?p=taxdetails&id=882015

MF680512

Pendanthura tinggiensis Chew et al. 2016: 232–238, figs. 2–8.

##### Materials

- **Type status:** Other material. **Location:** island: Mentinggi, Pulau Tinggi; country: Malaysia; stateProvince: Johor; verbatimCoordinates: N2°16'21.67", E104° 7'18.61"; **Event:** eventDate: 19 April 2013; habitat: Amongst coral rubble. Littoral 0.5 to 3 m depth; **Record Level:** collectionCode: (1 female) UKMMZ-1541; (1 male) UKMMZ-1542; (39 females) UKMMZ-1543; (2 males) UKMMZ-1544; (10 females) ZRC 2016.0013; (2 males) ZRC 2016.0014
- **Type status:** Other material. **Location:** island: Kg Pasir Panjang, Pulau Tinggi; country: Malaysia; stateProvince: Johor; verbatimCoordinates: N2°17'35.08", E104° 6'7.13"; **Event:** eventDate: 16 Aug 2012; habitat: Amongst coral rubble. Littoral 0.5 to 3 m depth; **Record Level:** collectionCode: (63 females, 3 males) UKMMZ-1544
- **Type status:** Other material. **Location:** island: Sebirah Kechil, Pulau Tinggi; country: Malaysia; stateProvince: Johor; verbatimCoordinates: N2°18.622', E104° 05.616'; **Event:** eventDate: 18 April 2013; habitat: Amongst coral rubble. Littoral 0.5 to 3 m depth; **Record Level:** collectionCode: (10 females) UKMMZ-1545
- **Type status:** Other material. **Location:** island: Kg Pasir Panjang, Pulau Tinggi; country: Malaysia; stateProvince: Johor; verbatimCoordinates: N2°17'35.08", E104° 6'7.13"; **Event:** eventDate: 15 June 2015; habitat: Amongst coral rubble. Littoral 0.5 to 3 m depth; **Record Level:** collectionCode: (37 females) UKMMZ-1546
- **Type status:** Other material. **Location:** island: Sebirah Kechil, Pulau Tinggi; country: Malaysia; stateProvince: Johor; verbatimCoordinates: N2°18.622', E104° 05.616'; **Event:** eventDate: 15 June 2015; habitat: Amongst coral rubble. Littoral 0.5 to 3 m depth; **Record Level:** collectionCode: (54 females) UKMMZ-1621

##### Distribution

Only known from type locality (Pulau Tinggi, Johor, Malaysia)

#### Pendanthura tiomanensis

Chew, bin Abdul Rahim & Mohd Yusof, 2016

DF907F7D-6A68-5B81-A3B9-B35B00D74847

http://www.marinespecies.org/aphia.php?p=taxdetails&id=882016

Pendanthura tiomanensis Chew et al. 2016: 238–241, figs. 9–11.

##### Materials

- **Type status:** Other material. **Location:** island: Batu Malang, Pulau Tioman; country: Malaysia; stateProvince: Pahang; verbatimCoordinates: N2°54'15.44", E104° 6'1.08"; **Event:** eventDate: 18 April 2014; habitat: Amongst coral rubble. Littoral 0.5 to 7 m depth; **Record Level:** collectionCode: (1 female) UKMMZ-1547; (3 females) UKMMZ-1549; (2 females) ZRC 2016.0015

##### Distribution

Only known from type locality (Pulau Tioman, Pahang, Malaysia)

#### Tinggianthura alba

Chew, bin Abdul Rahim & bin Haji Ross, 2014

62B3AE2C-BF38-5B25-B68D-2B7A33B78618

http://www.marinespecies.org/aphia.php?p=taxdetails&id=1054956

Tinggianthura alba Chew et al. 2014: 1–11, figs 2–9.

##### Materials

- **Type status:** Paratype. **Location:** island: Kampung Pasir Panjang, Pulau Tinggi; country: Malaysia; stateProvince: Johor; verbatimCoordinates: N2°17'35.08", E104° 6'7.13"; **Event:** eventDate: 16 August 2012; habitat: Amongst coral rubble. Littoral 0.5 to 3 m depth; **Record Level:** collectionCode: (12 females, 2 males, 1 juvenile) UKMMZ-1481; (12 females, 2 males, 1 juvenile) UKMMZ-1482; (12 females, 2 males, 1 juvenile) UKMMZ-1483
- **Type status:** Other material. **Location:** island: Kampung Pasir Panjang, Pulau Tinggi; country: Malaysia; stateProvince: Johor; verbatimCoordinates: N2°17'35.08", E104° 6'7.13"; **Event:** eventDate: 18 Dec 2012; habitat: Amongst coral rubble. Littoral 0.5 to 3 m depth; **Record Level:** collectionCode: (92 females, 8 males) UKMMZ-1622
- **Type status:** Other material. **Location:** island: Kampung Pasir Panjang, Pulau Tinggi; country: Malaysia; stateProvince: Johor; verbatimCoordinates: N2°17'35.08", E104° 6'7.13"; **Event:** eventDate: 28 Feb 2013; habitat: Amongst coral rubble. Littoral 0.5 to 3 m depth; **Record Level:** collectionCode: (26 females) UKMMZ-1623

##### Distribution

Only known from type locality (Pulau Tinggi, Johor, Malaysia)

#### Eisothistos besar

Müller 1992

1C60CB7B-7966-5A03-8094-CC89A0E23536

http://www.marinespecies.org/aphia.php?p=taxdetails&id=255500

Eisothistos besar Müller 1992c: 370–371, figs. 1–26.

##### Materials

- **Type status:** Holotype. **Location:** island: Pulau Babi Besar; country: Malaysia; stateProvince: Johor; verbatimCoordinates: N2°50.483', E104°9.566'; **Event:** habitat: Fringing reef, outer reef-flat, reef-margin and upper coral-slope; dead coral substratum, *Acropora* sp. and *Pocillopora damicornis*; **Record Level:** collectionCode: (immature adult, 1.7–2.1 mm) MHNG.

##### Distribution

Only known from type locality (Pulau Babi Besar, east coast Peninsular Malaysia)

##### Notes

This species record is based on literature only. No specimen was collected during the present study.

#### Eisothistos tiomanensis

Chew, bin Abdul Rahim & binti Mohd Yusof, 2018

E19DF907-90D4-5E2B-BE2F-052703486B5F

http://www.marinespecies.org/aphia.php?p=taxdetails&id=1056465

Eisothistos tiomanensis Chew et al. 2018: 76–79, figs. 5–6.

##### Materials

- **Type status:** Other material. **Location:** island: Labas, Pulau Tioman; country: Malaysia; stateProvince: Pahang; verbatimCoordinates: N2°53'13.71", E104° 3'54.65"; **Event:** eventDate: 18 April 2014; habitat: Amongst coral rubble. Littoral 0.5 to 15 m depth; **Record Level:** collectionCode: (1 male) UKMMZ-1559

##### Distribution

Only known from type locality (Pulau Tioman, Malaysia).

#### Expanathura collaris

(Kensley, 1979)

301CB3BA-E0F8-5CB3-94D1-2A3C59E4CE8D

http://www.marinespecies.org/aphia.php?p=taxdetails&id=260392

MF680511

Panathura collaris Kensley 1979: 823–827, figs 7–9; Kensley and Poore 1982: 635.
Expanathura collaris Wägele 1981: 89, 121–122; Negoescu 1999: 214–220, figs 9–11; Negoescu and Wägele 1984: 118; Negoescu and Brandt 2001: 121–129, figs 14–18; Poore and Lew Ton 2002: 26–32, figs 16–19, 20a.

##### Materials

- **Type status:** Other material. **Location:** island: Sebirah Kechil, Pulau Tinggi; country: Malaysia; stateProvince: Johor; verbatimCoordinates: N2°18.622’, E104° 5.616’; **Event:** eventDate: 16 May 2013; habitat: Amongst coral rubble, littoral 0.5 to 3 m; **Record Level:** collectionCode: (1 female) UKMMZ-1565; (1 Male) UKMMZ-1566; (54 females, 3 males) UKMMZ-1567
- **Type status:** Other material. **Location:** island: Mentinggi, Pulau Tinggi; country: Malaysia; stateProvince: Johor; verbatimCoordinates: N2°16’21.67", E104° 7’18.61"; **Event:** eventDate: 19 April 2013; habitat: Amongst coral rubble, littoral 0.5 to 3 m; **Record Level:** collectionCode: (12 females) UKMMZ-1568
- **Type status:** Other material. **Location:** island: Batu Malang, Pulau Tioman; country: Malaysia; stateProvince: Pahang; verbatimCoordinates: N2°54’15.44", E104° 6’1.08"; **Event:** eventDate: 18 April 2014; habitat: Amongst coral rubble, littoral 0.5 to 7 m; **Record Level:** collectionCode: (56 females, 9 males) UKMMZ-1569
- **Type status:** Other material. **Location:** island: Labas, Pulau Tioman; country: Malaysia; stateProvince: Pahang; verbatimCoordinates: N2°53’13.71", E104°3’54.65"; **Event:** eventDate: 18 April 2014; habitat: Amongst coral rubble, littoral 0.5 to 15 m; **Record Level:** collectionCode: (35 females, 2 males) UKMMZ-1570
- **Type status:** Other material. **Location:** island: Pantai Kok, Pulau Langkawi; country: Malaysia; stateProvince: Kedah; verbatimCoordinates: N6°21’56.05", E99°40’31.13"; **Event:** habitat: Intertidal coral rubble; **Record Level:** collectionCode: (14 females, 4 males) UKMMZ-1625
- **Type status:** Other material. **Location:** island: Batu Bonchek, Pulau Dayang; country: Malaysia; stateProvince: Johor; verbatimCoordinates: N2°28’40.90", E104° 30’19.12"; **Event:** eventDate: 26 July 2016; habitat: Amongst coral rubble, littoral 0.5 to 3 m; **Record Level:** collectionCode: (7 females, 1 male) UKMMZ-1568

##### Distribution

FIJI (Type locality); COOK ISLAND; MOOREA―Chesterfield and Melish Reefs, Coral Sea; LORD HOWE ISLAND―Tasman Sea; PAPUA NEW GUINEA; AUSTRALIA―Northern Territory, Queensland; MALAYSIA―Pulau Dayang, Pulau Tinggi, (New record); Pulau Tioman, Malaysia (New record); Pulau Langkawi (New record).

##### Notes

Fig. 2h

#### Kupellonura gidgee

Poore & Lew Ton, 1988

69F151D3-EB7F-5E87-A197-7ABCEC315E74

http://www.marinespecies.org/aphia.php?p=taxdetails&id=255527

Kupellonura gidgee Poore and Lew Ton 1988: 181, 183, 184, fig 10.

##### Materials

- **Type status:** Other material. **Location:** island: Batu Malang, Pulau Tioman; country: Malaysia; stateProvince: Pahang; verbatimCoordinates: N2°54'15.44", E104° 6'1.08"; **Event:** eventDate: 18 April 2014; habitat: Amongst coral rubble, littoral 0.5 to 7 m depth; **Record Level:** collectionCode: (1 female) UKMMZ-1602

##### Distribution

AUSTRALIA―Lizard Island (type locality); MALAYSIA―Pulau Tioman (new record).

##### Notes

Fig. 2i

#### Accalathura barnardi

(Nierstrasz, 1941)

E97A59EE-122C-5D8D-B6CC-368918E7A019

http://www.marinespecies.org/aphia.php?p=taxdetails&id=258455

Katanthura barnardi Nierstrasz 1941: 243–247, figs 1–13.
Accalathura barnardi Poore 1980: 59; Poore and Lew Ton 1990: 386, 388, fig 5.

##### Materials

- **Type status:** Other material. **Location:** island: Batu Malang, Pulau Tioman; country: Malaysia; stateProvince: Pahang; verbatimCoordinates: N2°54'15.44", E104° 6'1.08"; **Event:** eventDate: 18 April 2014; habitat: Amongst coral rubble, littoral 0.5 to 7 m depth; **Record Level:** collectionCode: (1 female) UKMMZ-1603; (1 male) UKMMZ-1604; (6 females) UKMMZ-1605
- **Type status:** Other material. **Location:** island: Labas, Pulau Tioman; country: Malaysia; stateProvince: Pahang; verbatimCoordinates: N2°53'13.71", E104° 3'54.65"; **Event:** eventDate: 18 April 2014; habitat: Amongst coral rubble, littoral 0.5 to 15 m depth; **Record Level:** collectionCode: (9 females, 2 males) UKMMZ-1606
- **Type status:** Other material. **Location:** island: Pulau Seri Buat; country: Malaysia; stateProvince: Pahang; verbatimCoordinates: N2°41'13.59", E103° 55'25.99"; **Event:** eventDate: 19 April 2014; habitat: Amongst coral rubble, littoral 0.5 to 7 m depth; **Record Level:** collectionCode: (6 females) UKMMZ-1607
- **Type status:** Other material. **Location:** island: Batu Bonchek, Pulau Dayang; country: Malaysia; stateProvince: Johor; verbatimCoordinates: N2°28'40.90", E104° 30'19.12"; **Event:** eventDate: 26 July 2016; habitat: Amongst coral rubble, littoral 0.5 to 3 m depth; **Record Level:** collectionCode: (13 females, 2 males) UKMMZ-1608
- **Type status:** Other material. **Location:** island: Teluk Rha, Pulau Aur; country: Malaysia; stateProvince: Johor; verbatimCoordinates: N2°28'17.24", E104° 30'53.14"; **Event:** eventDate: 27 July 2016; habitat: Amongst coral rubble, littoral 0.5 to 3 m depth; **Record Level:** collectionCode: (1 female, 1 males) UKMMZ-1609

##### Distribution

INDONESIA―Solo Strait, Java Sea (type locality); MALAYSIA―Pulau Tioman; Pulau Seri Buat (new record).

##### Notes

Fig. 2j

#### Accalathura borradailei

(Stebbing, 1904)

784B7E0C-E030-598C-942E-6D89B075715D

http://www.marinespecies.org/aphia.php?p=taxdetails&id=258457

MF680508

Calathura borradailei Gardiner 1904: 700, pl. 49A; Chilton 1924: 881.
Accalathura borradailei Barnard 1925: 149; Pillai 1966: 157–158, fig 3.

##### Materials

- **Type status:** Other material. **Location:** island: Kampung Pasir Panjang, Pulau Tinggi; country: Malaysia; stateProvince: Johor; verbatimCoordinates: N2°17'37.96", E104° 6'1.97"; **Event:** eventDate: 28 February 2013; habitat: coral rubble, intertidal; **Record Level:** collectionCode: (1 female) UKMMZ-1610; (1 male) UKMMZ-1611; (35 females, 3 males) UKMMZ-1612
- **Type status:** Other material. **Location:** island: Kampung Pasir Panjang, Pulau Tinggi; country: Malaysia; stateProvince: Johor; verbatimCoordinates: N2°17'37.96", E104° 6'1.97"; **Event:** eventDate: 18 December 2012; habitat: coral rubble, intertidal; **Record Level:** collectionCode: (34 females) UKMMZ-1613
- **Type status:** Other material. **Location:** island: Kampung Pasir Panjang, Pulau Tinggi; country: Malaysia; stateProvince: Johor; verbatimCoordinates: N2°17'37.96", E104°6'1.97"; **Event:** eventDate: 15 June 2015; habitat: coral rubble, intertidal; **Record Level:** collectionCode: (1 female) UKMMZ-1614
- **Type status:** Other material. **Location:** island: Sebirah Kechil, Pulau Tinggi; country: Malaysia; stateProvince: Johor; verbatimCoordinates: N2°18.622', E104° 05.616'; **Event:** eventDate: 15 June 2015; habitat: coral rubble, intertidal; **Record Level:** collectionCode: (20 females) UKMMZ-1615
- **Type status:** Other material. **Location:** island: Batu Bonchel, Pulau Dayang; country: Malaysia; stateProvince: Johor; verbatimCoordinates: N2°28'40.90", E104° 30'19.12"; **Event:** eventDate: 26 July 2015; habitat: coral rubble, 0.5 to 3 m depth; **Record Level:** collectionCode: (14 females, 1 male) UKMMZ-1616

##### Distribution

MALDIVES―Fadifolu (type locality); THAILAND; INDIA―Chilka Lake; Kollam; MALAYSIA―Pulau Dayang, Pulau Tinggi (new record).

##### Notes

Fig. 2k

#### Leptanthura coralliophila

Müller, 1992

AF83FC82-276E-54E6-A13F-60CB2DAE714A

http://www.marinespecies.org/aphia.php?p=taxdetails&id=255547

Leptanthura coralliophila Müller 1992a: 181–186, figs 1–19.

##### Materials

- **Type status:** Other material. **Location:** island: Pulau Seri Buat; country: Malaysia; stateProvince: Pahang; verbatimCoordinates: N2°41'13.59", E103°55'25.99"; **Event:** eventDate: 19 April 2014; habitat: Amongst coral rubble, intertidal to 7 m depth; **Record Level:** collectionCode: (1 female) UKMMZ-1617; (1 female) UKMMZ-1618

##### Distribution

MALAYSIA―Pulau Babi Besar (type locality); Pulau Seri Buat.

##### Notes

Fig. 2l

#### Paranthura astrolabium

Kensley, 1979

5C575653-D82F-5FF2-B70B-B83DF055C22D

http://www.marinespecies.org/aphia.php?p=taxdetails&id=255571

Paranthura astrolabium Kensley 1979: 830–833, figs. 12–13; Negoescu 1999: 237–242, figs 21–23.

##### Materials

- **Type status:** Other material. **Location:** island: Batu Malang, Pulau Tioman; country: Malaysia; stateProvince: Pahang; verbatimCoordinates: N2°54'15.44", E104° 6'1.08"; **Event:** eventDate: 18 April 2014; habitat: Amongst coral rubble, intertidal to 7 m; **Record Level:** collectionCode: (1 female) UKMMZ-1619; (18 females 1 male) UKMMZ-1621
- **Type status:** Other material. **Location:** island: Pulau Seri Buat; country: Malaysia; stateProvince: Pahang; verbatimCoordinates: N2°41'13.59", E103°55'25.99"; **Event:** eventDate: 19 April 2014; habitat: Amongst coral rubble, intertidal to 7 m; **Record Level:** collectionCode: (3 females) UKMMZ-1622

##### Distribution

FIJI―Great Astrolabe Barrier Reef (type locality); Suva Reef; MALAYSIA―Pulau Tioman, Malaysia; Pulau Seri Buat (new record).

##### Notes

Fig. 2m

#### Paranthura setigera

Negoescu, 1997

3282B05D-7D7F-5C46-B4E7-00DDFA5AFFD7

http://www.marinespecies.org/aphia.php?p=taxdetails&id=255615

Paranthura setigera Negoescu 1997: 232–241, figs. 33–38.

##### Materials

- **Type status:** Other material. **Location:** island: Kampung Pasir Panjang, Pulau Tinggi; country: Malaysia; stateProvince: Johor; verbatimCoordinates: N2°17'37.96", E 104° 6'1.97"; **Event:** eventDate: 18 December 2012; habitat: coral rubble, intertidal; **Record Level:** collectionCode: (1 female) UKMMZ-1623; (1 male) UKMMZ-1624; (91 females, 7 males) UKMMZ-1625
- **Type status:** Other material. **Location:** island: Kampung Pasir Panjang, Pulau Tinggi; country: Malaysia; stateProvince: Johor; verbatimCoordinates: N2°17'37.96", E104° 6'1.97"; **Event:** eventDate: 28 Feb 2013; habitat: coral rubble, intertidal; **Record Level:** collectionCode: (26 females) UKMMZ-1626

##### Distribution

INDONESIA―Southeast Bali Island, Sanur beach, Bali Island (type locality); MALAYSIA―Pulau Tinggi (new record).

##### Notes

Fig. 2n

#### Paranthura seychellensis

Kensley & Schotte, 2000

A15F5BC0-FD4C-5880-BEFB-5FED3C68858D

http://www.marinespecies.org/aphia.php?p=taxdetails&id=211339

Paranthura seychellensis Kensley and Schotte 2000: 2114–2116, fig 39.

##### Materials

- **Type status:** Holotype. **Location:** island: Labas, Pulau Tioman; country: Malaysia; stateProvince: Pahang; verbatimCoordinates: N2°53'13.71", E104° 3'54.65"; **Event:** eventDate: 18 April 2014; habitat: Amongst coral rubble, littoral 0.5 to 15 m depth; **Record Level:** collectionCode: (1 female) UKMMZ-1627; (1 male) UKMMZ-1628; (1 premature female) UKMMZ-1629
- **Type status:** Holotype. **Location:** island: Labas, Pulau Tioman; country: Malaysia; stateProvince: Pahang; verbatimCoordinates: N2°53'13.71", E104° 3'54.65"; **Event:** eventDate: 18 April 2014; habitat: Amongst coral rubble, littoral 0.5 to 15 m depth; **Record Level:** collectionCode: (1 female, 4 males) UKMMZ-1630

##### Distribution

SEYCHELLES―Mahé Beach Mahé Island (type locality); MALAYSIA―Pulau Tioman (new record).

##### Notes

Fig. 2o

## Discussion

Currently five families of the marine Anthuroidea are known from the Malaysian Peninsular, comprising 12 genera and 24 species. Of these, 10 species (*Apanthura bruscai, A. tiomanae, Mesanthura asiatica, M. kiliani, Pendanthura tinggiensis, P. tiomanensis, Tinggianthura alba, Eisothistos besar, E. tiomanensis* and *Leptanthura coralliophila*) are recently discovered Malaysian species and they contribute about 1.5% of all known Anthuroidea of the world. Clearly, this number is only a fraction of their true diversity, given that habitats like shallow reefs, seagrasses and mangroves have not received much taxonomic attention. More species are yet to be discovered, especially after exploring inaccessible habitats, revealing of cryptic species using molecular methods and exploring east Malaysian (Sabah and Sarawak) waters. The latter is a region of high known marine biodiversity.

The anthuroids of Peninsular Malaysia are similarly taxonomically represented as the adjacent thoroughly studied Indian Ocean region by Kensley and Schotte (2000). In terms of faunistic composition, both the regions show a high proportion of Anthuridae species with 58.3% in the Malaysian region and 45.8% in the Indian Ocean region. It is followed by a small number of Leptanthuridae (12.5% in Malaysia and 20.8% in the Indian Ocean), Paranthuridae (12.5% in Malaysia and 12.5% in the Indian Ocean), Expanathuridae (12.5% in Malaysia and 8.3% in the Indian Ocean) and Hyssuridae (4.2% in Malaysia and 8.3% in the Indian Ocean). Having highlighted the similarity, a minor but crucial difference was also noticed, particularly on the occurrence of Antheluridae species in the Indian Ocean. According to Kensley (2001), most of the Antheluridae species are restricted to deep-water, cool-temperate or polar regions. Although several species are found to inhabit the warmer and shallower seas, particularly the eight species of *Anthomuda* Schultz, 1979, they are mostly captured from the Indian Ocean (Poore and Lew Ton 1988). The high species diversity, particularly in the family Anthuridae (17 species), can be attributed to the fact that the family is the largest and most widespread (Chew et al. 2014, Song and Min 2015). Some of its species-rich genera, *Apanthura* and *Mesanthura*, are recorded with the highest species diversity amongst the Malaysian anthuroid fauna. On the other hand, there is no record of any Antheluridae species occurring in the Malaysian region, most probably due to the fact that they are rare in the warmer seas (Kensley 2001, Poore and Lew Ton 1988).

Currently, all known Malaysian anthuroids are found to be associated with coral rubble. Other habitats, such as sandy and muddy sediments and algal beds, have not been sampled. Due to the fact that they are very well represented in the high spatial complexity coral rubble (Richmond 2011, Kensley 1998), coral rubble samplings were prioritised in the past works, as well as in the present study. From all coral rubble collections around the world, one generally true characteristic is that the *Eisothistos* species are rare and, if reported, they have usually been collected from breaking open serpulid tubes (Wägele 1981). Moreover, *Amakusanthura koonyumae*, a species previously known to inhabit sandy substratum (Bamber 1997, Bamber 2008), is now found to be associated with coral rubble of the Malaysian coral reefs.

## Supplementary Material

XML Treatment for Amakusanthura koonyumae

XML Treatment for Apanthura bruscai

XML Treatment for Apanthura pariensis

XML Treatment for Apanthura stocki

XML Treatment for Apanthura tiomanae

XML Treatment for Cyathura bentotae

XML Treatment for Mesanthura albolineata

XML Treatment for Mesanthura asiatica

XML Treatment for Mesanthura kiliani

XML Treatment for Mesanthura protei

XML Treatment for Mesanthura quadrata

XML Treatment for Pendanthura tinggiensis

XML Treatment for Pendanthura tiomanensis

XML Treatment for Tinggianthura alba

XML Treatment for Eisothistos besar

XML Treatment for Eisothistos tiomanensis

XML Treatment for Expanathura collaris

XML Treatment for Kupellonura gidgee

XML Treatment for Accalathura barnardi

XML Treatment for Accalathura borradailei

XML Treatment for Leptanthura coralliophila

XML Treatment for Paranthura astrolabium

XML Treatment for Paranthura setigera

XML Treatment for Paranthura seychellensis

## Figures and Tables

**Figure 2.**
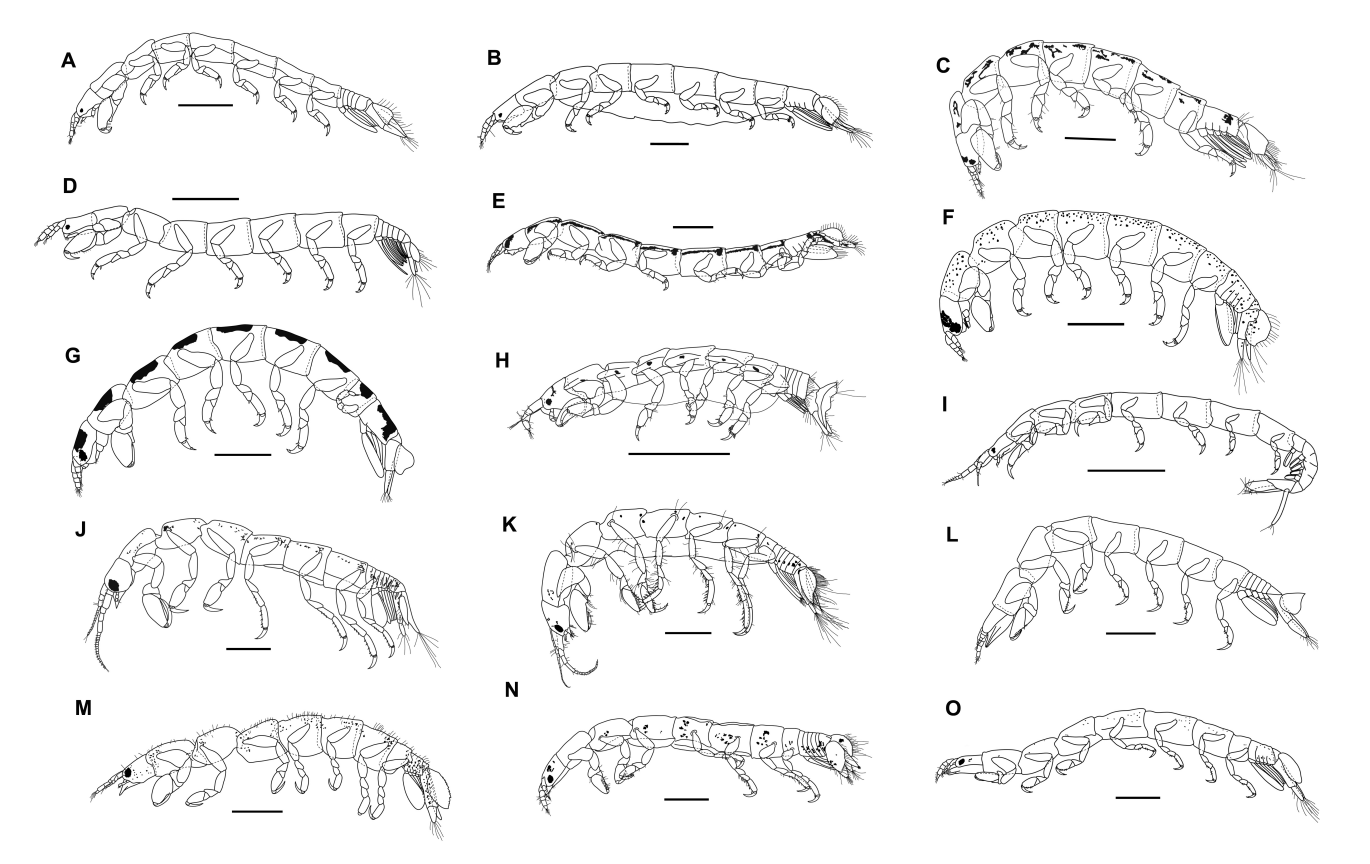
Habitus of Anthuroidea (females) of the reefs of Peninsular Malaysia: **A.** *Amakusanthura koonyumae* Bamber, 1997; **B.** *Apanthura pariensis* Negoescu, 1997; **C.** *Apanthura stocki* (Müller, 1991); **D.** *Apanthura tiomanae* Müller, 1992; **E.** *Mesanthura asiatica* Müller, 1993; **F.** *Mesanthura protei* Kensley, 1980; **G.** *Mesanthura quadrata* Kensley & Schotte, 2000; **H.** *Expanathura collaris* (Kensley, 1979); **I.** *Kupellonura gidgee* Poore & Lew Ton, 1988; **J.** *Accalathura barnardi* (Nierstrasz, 1941); **K.** *Accalathura borradailei* (Stebbing, 1904); **L.** *Leptanthura coralliophila* Müller, 1992; **M.** *Paranthura astrolabium* Kensley, 1979; **N.** *Paranthura setigera* Negoescu, 1997; **O.** *Paranthura seychellensis* Kensley & Schotte, 2000. All species are illustrated here for the first time.
